# Subgroups of older adults with osteoarthritis based upon differing comorbid symptom presentations and potential underlying pain mechanisms

**DOI:** 10.1186/ar3449

**Published:** 2011-08-24

**Authors:** Susan L Murphy, Angela K Lyden, Kristine Phillips, Daniel J Clauw, David A Williams

**Affiliations:** 1 Department of Physical Medicine and Rehabilitation, University of Michigan, 300 North Ingalls, 9th floor, Ann Arbor, MI 48109-2007, USA; 2 Geriatric Research, Education and Clinical Center (GRECC), Veterans Affairs Ann Arbor Health Care System, Michigan. 2215 Fuller Rd, Ann Arbor, MI 48105, USA; 3 Division of Rheumatology, Department of Internal Medicine, University of Michigan, Domino's Farms, 24 Frank Lloyd Wright Drive, Lobby M, Ann Arbor, MI 48105-5737, USA; 4 Departments of Anesthesiology and Psychiatry, University of Michigan, Domino's Farms, 24 Frank Lloyd Wright Drive, Lobby M, Ann Arbor, MI 48105-5737, USA; 5 Department of Psychology, University of Michigan, Domino's Farms, 24 Frank Lloyd Wright Drive, Lobby M, Ann Arbor, MI 48105-5737, USA

## Abstract

**Introduction:**

Although people with knee and hip osteoarthritis (OA) seek treatment because of pain, many of these individuals have commonly co-occurring symptoms (for example, fatigue, sleep problems, mood disorders). The purpose of this study was to characterize adults with OA by identifying subgroups with the above comorbid symptoms along with illness burden (a composite measure of somatic symptoms) to begin to examine whether subsets may have differing underlying pain mechanisms.

**Methods:**

Community-living older adults with symptomatic knee and hip OA (*n *= 129) participated (68% with knee OA, 38% with hip OA). Hierarchical agglomerative cluster analysis was used. To determine the relative contribution of each variable in a cluster, multivariate analysis of variance was used.

**Results:**

We found three clusters. Cluster 1 (*n *= 45) had high levels of pain, fatigue, sleep problems, and mood disturbances. Cluster 2 (*n *= 38) had intermediate degrees of depression and fatigue, but low pain and good sleep. Cluster 3 (*n *= 42) had the lowest levels of pain, fatigue, and depression, but worse sleep quality than Cluster 2.

**Conclusions:**

In adults with symptomatic OA, three distinct subgroups were identified. Although replication is needed, many individuals with OA had symptoms other than joint pain and some (such as those in Cluster 1) may have relatively stronger central nervous system (CNS) contributions to their symptoms. For such individuals, therapies may need to include centrally-acting components in addition to traditional peripheral approaches.

## Introduction

Osteoarthritis (OA) is the leading cause of disability in US adults and its prevalence is expected to double by 2020 [1]. Historically, the "disease" of OA has been viewed primarily as damage to the cartilage and bone. As such, the magnitude of damage or inflammation of these structures is often associated with higher symptom levels. Population-based studies suggest otherwise; 30 to 50% of individuals with moderate to severe radiographic changes of OA are asymptomatic, and approximately 10% of individuals with moderate to severe knee pain have normal radiographs [2,3]. Psychosocial factors do account for some of this variance in pain and other symptoms, but only modestly [4-6]. There also may be other bone, joint, or physical changes associated with symptom severity which are still not well-understood. The current failure of peripheral damage, inflammation, or other factors to explain the presence, absence, or severity of chronic pain suggests the need to identify additional salient factors that may be contributing to the experience of OA.

There is growing evidence in OA that there is a central component to pain. Recent studies of animal models provide support for central sensitization of nociceptive pathways [7,8]. In addition, focus groups identified a subset of patients with chronic, symptomatic knee OA who used pain quality descriptors that were suggestive of neuropathic pain [9]. There are several studies suggesting that OA patients display diffuse hyperalgesia to mechanical or heat stimuli (that is, suggestive of central nervous system (CNS) mediation) [10-12]. Kosek demonstrated that individuals with hip OA had reduced descending analgesic activity, which partially normalized following hip arthroplasty, suggesting the involvement of central factors influencing the activity of peripheral nociceptive input [13]. Gwilym and colleagues used both experimental pain testing and more sophisticated functional neuroimaging procedures to show evidence of augmented CNS processing of pain in 20 OA patients [14]. In a separate study, this same group showed that atrophy of the thalamus was seen at baseline on OA and improved following arthroplasty [15], again underscoring the role of the CNS in OA pain. Finally, recent randomized controlled trials have demonstrated that compounds that alter pain neurotransmitters centrally, such as serotonin and norepinephrine, (for example, duloxetine, tricyclics), are efficacious in OA [16,17]. In aggregate, these studies do not imply that peripheral factors are unimportant in OA; rather, peripheral factors alone are insufficient to account for symptoms in some or many individuals with OA. Whether or not CNS augmentation plays a prominent role in OA pain is likely to be tied to genetic predisposition, environmental stressors, and the degree of illness burden a given person is experiencing at the time [18-25]. Non-region-specific symptoms accompanying pain, such as fatigue, cognitive problems, sleep problems, and perturbations of mood, are systemically-mediated symptoms that may index more central involvement in the maintenance of illnesses, such as pain [24,26,27]. These symptoms may be important to target treatment in addition to pain. For instance, in a previous study with an OA sample, fatigue was actually more related to functional disability than to pain [28]. To optimize treatment, it may be necessary to better characterize people with OA and determine if there are subsets of people who have symptom presentations that may reflect different underlying pain mechanisms. Since OA treatment is still largely focused on alleviating pain at the peripheral site (for example, strength training to reduce knee joint stress), it is particularly important to examine if there is a subgroup of people with OA that has a symptom cluster supporting a clinical presentation of centrally-mediated pain. For those people, it may be important to focus on treatment that is more commonly used in centrally-mediated pain conditions, (such as behavioral treatments and lifestyle interventions). Classification of people into empirically-derived subgroups has been done in other chronic pain conditions, such as fibromyalgia and low back pain, based upon symptoms and psychosocial factors and has successfully identified subgroups of patients with distinct phenotypic characterization and with differential response to treatment [29-31]. OA is similar to these other chronic pain conditions in that many patients experience multifocal pain, fatigue, sleep disturbances, and mood disorders in addition to joint pain; however, no studies have examined if there are distinct subgroups of people with OA who differ based on their symptomatology.

The purpose of this study was to identify specific subgroups of individuals who initially presented with symptomatic knee or hip OA. Subgroups would be based upon levels of comorbid centrally-mediated symptomatology, such as fatigue, sleep disturbance, mood, as well as other symptoms, which we refer to as illness burden, known to occur in other centrally-mediated pain states (for example, irritable bowel syndrome, temporomandibular joint disorder, interstitial cystitis, and so on) [32]. We hypothesized that one subgroup would appear to represent OA patients who predominantly have joint pain, whereas other subgroups would have different symptom presentations that support differential manifestations of CNS-mediated symptomatology.

## Materials and methods

### Study design, participants, and setting

Our primary study was designed to examine the relationship among pain, fatigue, and physical activity in older adults with osteoarthritis. Potential participants were recruited in various ways: through fliers (in clinics at the University of Michigan Hospital System, at the Ann Arbor VA Health Care System, and around several cities in southeastern Michigan), newspaper advertisements, and online through the University of Michigan's clinical subjects website. All individuals first signed an informed consent document outlining the research project; this was approved by the University of Michigan Institutional Review Board and Ann Arbor Veteran's Affairs Human Subjects Review Board. The individuals underwent a comprehensive screening evaluation with a geriatrics nurse practitioner who was trained by a rheumatologist to ascertain the presence of clinical knee or hip OA using the American College of Rheumatology (ACR) clinical criteria [33,34] and to evaluate for the presence of comorbidities. A total of 129 community-dwelling adults participated; they were aged 65 years and older who initially self-reported knee or hip OA.

### Inclusion and exclusion criteria

To recruit people with painful OA, individuals were included if they reported pain in a joint with OA on the Western Ontario and McMaster Universities Osteoarthritis Index (WOMAC) scale (Likert version) of ≥ 4 with at least two of the five items on the scale rated as moderate pain or more [35]. This criterion was chosen because our primary study involved an examination of the relationship of pain and fatigue to physical activity in a home monitoring period, and we felt that this criterion would allow us to capture variability in symptoms over that period. In addition, participants were included if they reported experiencing fatigue symptoms at least a moderate amount of the time (that is, three to four of the past seven days) from one of two questions from the Center for Epidemiologic Studies Depression Scale (CES-D; How often in the past week: Did you feel like everything you did was an effort? or Could you not get going?) [36]. Participants also needed to have adequate cognition and be able to see, hear and operate the accelerometer used for pain reporting in the study. Individuals were excluded if: 1) they reported other medical conditions that were capable of causing fatigue (acute illnesses or exacerbations of chronic illnesses, including common viral or bacterial infections, autoimmune diseases, fibromyalgia, chronic fatigue syndrome, and any uncontrolled illness); 2) they were undergoing cancer treatment or had received treatment for cancer in the previous 12 months; 3) they reported doctor-diagnosed obstructive sleep apnea; 4) they presented with untreated anemia or thyroid disorders per blood work; or 5) they were non-ambulatory.

### Measures

#### Pain

Individuals were provided with a wrist-worn accelerometer (Actiwatch-Score, Philips Respironics-Mini-Mitter, Bend, OR, USA) that measured physical activity patterns and allowed ecological momentary assessment of pain five times a day over a five-day home monitoring period (that is, Monday through Friday). Based on our experience in past studies, we chose sampling times that represented each part of the day (wake-up, late morning to 11 am, afternoon to 3 pm, evening to 7 pm, and bedtime). After being prompted by the device's audible alarm, participants rated their pain on a scale of 0 to 10 ("No pain at all" to "Pain as bad as I can imagine"). Pain ratings were then summed over each day and averaged across all days to derive an average pain rating. Because of our interest in multi-focal pain mechanisms, we chose to include this measure of pain rather than the WOMAC pain subscale as we think it better captures global pain experience compared to a disease-specific instrument.

#### Mood

Depressive symptomatology was assessed using the Center of Epidemiologic Studies Depression Scale (CES-D) [36]. A score of 16 or greater on the CES-D has been associated with clinically significant depressive symptoms [37].

#### Fatigue

The Brief Fatigue Inventory (BFI) was used to measure total fatigue [38] calculated by averaging ratings on nine items in which participants rate fatigue severity ("No fatigue" to "As bad as you can imagine") and interference ("Does not interfere" to "Completely interferes") in daily life over the last week on a scale of 0 to 10 [38].

#### Illness burden

Illness burden is a concept that reflects the self-reported symptom load experienced by an individual. For this study, participants were asked whether they experienced any of 41 different somatic symptoms with the sum representing the "illness burden" variable in the analysis. Examples of the types of symptoms include: stomach pains, headaches, cognitive dysfunction, muscle weakness or stiffness, restless sleep, and daytime sleepiness. Individually these symptoms may represent various health problems associated with different body systems, but in aggregate they may be viewed as the amount of burden a given individual experiences stemming from centrally-mediated somatic symptoms, such as fibromyalgia, irritable bowel syndrome, and chronic fatigue syndrome. In other chronic pain conditions, greater endorsement of such symptoms has been associated with greater central pain and dysfunction status [39-41].

#### Sleep problems

Sleep problems were assessed using the Pittsburgh Sleep Quality Index (PSQI) [42]. A total score is calculated from seven subscales. A score of 5 or more indicates greater difficulties with sleep.

#### Functional status

Functional status measures were not included in clustering, but were of interest to determine how different symptom clusters were associated with function, included both the short-form WOMAC physical disability scale [43], and the objective "timed up and go test" [44], which measures the time (in seconds) to get up from a chair, walk 20 feet and return to the chair.

### Statistical analysis

The variables selected for analytic clustering were chosen based upon their known association with other central pain conditions (for example, fibromyalgia and irritable bowel syndrome) and by virtue of these variables being of centrally-mediated origin rather than being symptoms of peripheral or localized etiology. The symptoms chosen for clustering included the following: pain, fatigue, sleep problems, depressive symptoms, and somatic symptoms (representing overall illness burden). Hierarchical agglomerative cluster analysis utilized Ward's method with Squared Euclidean Distances in the proximities matrix [45]. Visual inspection of the dendogram and an evaluation of the dissimilarity measure were used to identify a three-cluster solution as being the best fit for these data. Each variable within the clustering solution was included in a subsequent multivariate analysis of variance (MANOVA) comparing the three clusters in order to determine the relative contribution of each variable between clusters [46]. To facilitate grouping individuals into clusters, a follow-up discriminant analysis was performed. A constant and set of five coefficients (that is, one for each variable in the analysis) were derived for each cluster. These functions also facilitate accounting for the variance attributable to each cluster and the degree of separation between functions. Additional analyses of variance (ANOVA) were used to identify concurrent clinical and demographic characteristics that differed significantly depending upon subgroup membership. All analyses were completed using PASW v18.

## Results

### Participant characteristics

Characteristics of the sample are shown in Table 1. There were 172 people eligible for this analysis from 605 people who underwent an initial phone screening for the study. Of the 605, 27% were screened out due to not having enough pain, 15% were screened out due to not having enough fatigue, 12% were not aged 65 and older, 9% had other conditions (for example, obstructive sleep apnea, rheumatoid arthritis, inadequate cognition), and 2% had a diagnosis of fibromyalgia. Complete data were available on 129 of the 172 participants who were screened. The missing data were from participants who decided not to participate or were ineligible after full evaluation of the screening results (such as due to not meeting the clinical criteria for OA or abnormal results from blood work). Therefore, these individuals did not complete the home monitoring period required to obtain the average five-day pain rating. Compared to the 129 participants (shown in Table 1), the 43 participants had less pain on the WOMAC scale (6.7 ± 3.7, *P *= .06) and less physical disability on the short-Form WOMAC scale (10.83/28 ± 4.26, *P *= .07). These participants had similar levels of fatigue on the BFI (4.4 ± 1.9, *P *= .91) and depression on the CES-D (12.2 ± 7.9, *P *= .97) compared to the sample of 129 people. A total of 79 females and 50 males were included in the current analysis with the majority of the sample identifying themselves as either Caucasian (87%) or African American (9%). Knee OA was the most common diagnosis (69%), followed by both knee and hip OA (11%), and hip OA alone (20%). Scores on the WOMAC indicated a mild-moderate pain experience among participants (8.5 ± 3.2) with overall moderate levels of self-reported physical dysfunction (12.30 ± 4.67). Average pain severity for the sample was 3.06 (1.62) and average fatigue severity was 3.77 (1.67).

**Table 1:** Characteristics of the entire study sample (*n *= 129, 79 female, 50 male)

Variable		Mean (SD)	Range
Age (years)		72.2 (9.8)	65 to 90
BMI (kg/m^2^)		30.5 (5.9)	21.5 to 49.9
Self-reported race	Caucasian	112 (86.8%)	
African American		12 (9.3%)	
Asian		2 (1.6%)	
More than one		1 (.8%)	
Chose not to report		2 (1.6%)	
Study joint	knee	88 (68.2%)	
hip		41 (31.7%)	
% Veterans		26/120 (20.2%)	
WOMAC pain		7.9 (3.4)	2 to 20
WOMAC stiffness		3.3 (1.7)	0 to 8
WOMAC disability		20.9 (10.3)	3 to 42
BFI total		4.5 (2.0)	0.25 to 8.75
Self-reported duration of pain (months)		132.1 (146.5)	0 to 708

### Cluster analysis

Three clusters were identified as being the best fit for these data. Significant separation of the variables contained within the clustering solution was confirmed overall by MANOVA (Wilks' λ = .148, F(10,236) = 37.69, *P *< .0001). Subsequent univariate ANOVAs indicated significant differences were observed between clusters for all variables making up the clusters (all *P *≤.0001). *Post-hoc *analyses revealed significant differences between all variables and all pairs of clusters except for pain severity where there was not a significant difference between Clusters 2 and 3 (*P *= .09). Table 2 shows the characteristics of each cluster by variable.

**Table 2:** Cluster characteristics^a^

Variable	Cluster 1 (*n *= 45)	Cluster 2 (*n *= 38)	Cluster 3 (*n *= 42)
CES-D-depression	17.3 (7.1)	9.9 (5.0)	5.0 (3.4)
BFI-fatigue	6.2 (1.4)	4.0 (1.5)	3.0 (1.4)
PSQI	10.6 (3.6)	5.5 (2.1)	7.2 (2.9)
Average pain severity	3.9 (1.6)	2.3 (1.0)	2.9 (1.7)
No. of symptoms - Illness burden	12.2 (2.7)	10.4 (2.9)	5.3 (3.6)

^a^Values are the mean (SD). Multivariate ANOVA confirmed that each variable was differentiated by the cluster solution (Wilks' λ = .148, F(10,236) = 37.69, *P *< .0001) and univariate ANOVAs confirmed that each variable significantly differentiated the clusters (all *P *< .0001).

Cluster 1 comprised 36% of the sample (*n *= 45). This group had the most pathological scores on all clustering variables compared to the other two groups and was characterized by the highest level of depressive symptoms (17.3 which is above the clinical cut-point of 16), highest fatigue and average pain (6.2 and 3.9/10 respectively), the worst sleep, and the greatest illness burden (endorsing 30% of somatic symptoms that were queried).

Cluster 2 comprised 30% of the sample (*n *= 38). The group was characterized by subclinical levels of depression and moderate fatigue, but had low pain and lower levels of sleep problems (that is, 5.5). They had a moderate illness burden (endorsing 25% of somatic symptoms).

Cluster 3, comprised 34% of the sample (*n *= 42). This group had clinically-relevant levels of sleep problems, but mild levels of average pain (that is, 2.9/10). The group had the lowest levels of fatigue and depression, and markedly low levels of illness burden compared to the two other clusters (that is, 13% of somatic symptoms endorsed).

### Clinical and demographic variables among subgroups

Age did not differ across clusters (mean = 72 years; F(2,122) = .036, *P *= .97), and there was no significant relationship between gender and cluster assignment (χ^2 ^= 3.72, *P *= .16). Similarly, body mass index (BMI) did not differ across clusters, being within the category of obese (for example, 30 to 31) on average (F(2,122) = 1.02, *P *= .36). With regard to OA joints per the ACR clinical criteria, we examined the clusters according to whether they had knee OA alone or whether OA was in both the hip and knee or in the hip joints alone. The most prevalent group in all clusters had knee OA alone (82% in Cluster 1, 64% in Cluster 2, and 63% in Cluster 3); however, location of OA joint was not significantly different across clusters (χ^2 ^= 3.86; *P *= .15). Duration of OA pain was not significantly different across the groups and there was a large amount of variability across people (F(2,122) = 2.58, *P *= .08); although Cluster 2 had the longest pain duration at 177 months (± 180) compared to Cluster 1 and Cluster 3 (124 ± 120 and 105 ± 126).

Differences in clusters were found in stiffness, physical disability, and objective physical function. Stiffness on the WOMAC scale was significantly different across groups (F(2,121) = 5.95, *P *= .003), specifically between Cluster 1, which had the highest level of stiffness (3.91 ± 1.60), and Cluster 3, which had the lowest level (2.74 ± 1.70). Physical disability on the WOMAC disability scale was also significantly different across clusters (F(2,122) = 16.18, *P *= .0001). The significant difference in means from *post-hoc *testing was 14.84 ± 4.50 in Cluster 1 compared to Clusters 2 and 3 (9.84 ± 3.84 and 11.27 ± 3.98 respectively). On the timed up and go test, Cluster 1 had a mean of 13.5 (± 8.9) which is a cut-off score denoting fall risk (43) and was significantly different from Clusters 2 and 3 which were similar in their performance on this test (10.5 ± 2.1 and 10.2 ± 2.3 respectively).

### Discriminant functions

**Figure 1:**
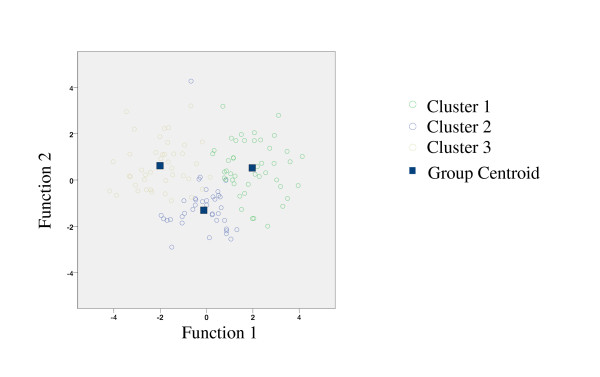
**Illustration of required canonical discriminant functions to differentiate Clusters 1, 2 and 3**. Two discriminant functions were identified that significantly differentiated the clusters accounting for 71% and 29% of the variance among them (Wilk's Lambda for Function 1: χ2 = 168.28, *P *< .0001; and for Function 2: χ2 = 59.10, *P *< .0001).

Two discriminant functions (that is, linear combinations of the independent variables) were identified that significantly differentiated the clusters accounting for 71% and 29% of the variance among them (Wilk's Lambda for Function 1: χ2 = 168.28, *P *< .0001; and for Function 2: χ2 = 59.10, *P *< .0001). The first function was weighted towards reported fatigue and number of endorsed somatic symptoms, while the second function was weighted toward reported pain and sleep quality. Figure 1 shows the multivariate group means (called the group centroids) for the three clusters, the spread of the data in each cluster (represented by the different color circles), and the discriminant functions. The distance between the centroids and amount of overlap between the circles of each cluster shows how the clusters are differentiated. The first discriminant function on the x axis separates Cluster 3 from Cluster 1, while the second function on the y axis is needed to differentiate Cluster 2 from the other two. As a second step, we predicted group membership using a constant and coefficients for each of the predictor variables. These values were applied in the same manner as for a regression where the coefficient is multiplied by the individual value and all values are summed. A value was generated for each cluster and the highest value of the three represents the cluster assignment. Table 3 shows the coefficients for each cluster and illustrates an example of the predictive process. Based on this method, we correctly classified 95.2% of participants in the sample into their empirically derived clusters.

**Table 3:** Discriminative analysis of cluster characteristics^a^

Variable	Cluster 1	Cluster 2	Cluster 3
Constant	-27.56	-13.30	-8.72
CES-D	.49	.30	.09
BFI	**2.89**	**1.93**	**1.23**
Sleep disturbance	**1.01**	.40	.88
Pain	.85	.17	**1.20**
Illness burden	**1.04**	**1.05**	.27

^a^Values are the coefficients for discriminant functions for each cluster. Values in boldface indicate the variable loads highest for that particular cluster.

## Discussion

In this study, we were interested in characterizing a sample of older adults with symptomatic knee and hip OA. Three subgroups provided the best fit to our cluster analytic model and each had different symptom presentations that may be reflective of different degrees and types of CNS-mediated symptomatology. There was a group (Cluster 1; 36% of the sample) that appeared to have symptoms reminiscent of fibromyalgia, including the highest ratings on both pain and fatigue, as well as the worst ratings on depressive symptoms and sleep. In addition, this group also had the highest illness burden (that is, the number of somatic symptoms endorsed which are common in other centrally-mediated pain states, such as irritable bowel syndrome, temporomandibular joint disorder, and interstitial cystitis), further supporting that a potential CNS contribution to symptoms.

Cluster 2 had intermediate levels of depressive symptoms and modest levels of other somatic symptoms, suggesting these individuals were suffering from mild depression. Cluster 2 appears to be a group of relatively mixed central influence, with perhaps mild depressive symptomatology, but no significant evidence of sensory amplification symptoms that are more classically seen in fibromyalgia and related conditions. Cluster 3 had low levels of all symptoms except difficulties with sleep, where they had slightly greater symptomatology than Cluster 2. Individuals within Cluster 3 may represent those individuals who have been traditionally thought of as having OA confined to the joint. With the exception of sleep problems, these individuals showed minimal evidence of known central factors to be influencing the overall condition.

Although our data appear to support the idea that differences in symptom presentation among clusters may be due to differences in pain mechanisms, potential alternative explanations were considered. For example, clusters may have been differentiated due to differences in pain severity rather than differences in pain mechanism. However, because all groups have mild-moderate pain (2.3 to 3.9), this seems unlikely. In addition, it could be argued that the differences among clusters may be due to physical function. Specifically, Cluster 1, the group that was most symptomatic, also had significantly worse scores on the WOMAC physical disability scale and on the timed up and go test than members of the other two clusters. This finding is consistent with known associations between symptoms, depression, and function in chronic pain [47,48], and reduced physical function could be a manifestation of having a high symptom burden. However, a longitudinal study would be needed to examine causality. In an additional analysis, we further explored the relationship between pain severity and physical function by examining correlations by cluster. Correlations between pain severity and the physical function variables (WOMAC physical disability and the timed up and go test) were strongest and only statistically significant for Cluster 3 (r = .55 and .32 respectively) suggesting that this cluster, the one thought to be more of a traditional OA group with joint pain and few other symptoms, has activity-related pain.

There is a growing body of literature that suggests pain experienced in some individuals with OA could be due to sensitization of both local and central pain pathways [49,50]. Central pain disorders, such as fibromyalgia, are characterized by widespread pain and the presence of symptoms, such as fatigue, sleep problems, mood disorders, and overall high illness burden, that were found in participants in Cluster 1. This is interesting in light of the fact we specifically excluded individuals with a diagnosis of fibromyalgia from participating in the study, which was further confirmed by ascertaining the presence of widespread pain using a modification of the 1990 ACR criteria [51]. Although we did not perform a tender-point exam, we did ask individuals about the presence of pain in each of the four body quadrants, as well as the axial skeleton, and about tenderness to pressure at different points on the body. Individuals needed to answer in the affirmative to each of the widespread pain questions to be excluded under the 'fibromyalgia' criterion. These findings suggest that treatment approaches may need to be extended beyond the joint in people with OA who present like those in Cluster 1. Similar to another study [52], this study provides support for the idea that OA is likely a "mixed pain state," with individual variability in the relative balance of peripheral (that is, nociceptive) and central elements of pain. Although it is impossible to determine from our current data, it is likely that some participants might have undiagnosed fibromyalgia or widespread pain as defined by the latest fibromyalgia diagnostic criteria [53]. This possibility bolsters the idea that there is a need for a broader and more flexible approach to diagnosis and treatment of symptoms in people who have OA. Potentially such individuals may benefit from treatments that target more centrally acting mechanisms and that are appropriate for the management of fatigue, sleep, and depression, and might be expected to preferentially respond to drugs such as duloxetine, which has recently been shown to be effective in OA [16].

These findings should be considered preliminary given that the clustering solution still requires replication in another sample of OA patients. In addition, this study was a first step into examining pain mechanisms in an OA sample using symptom presentation as a grouping variable. More sophisticated measurement, such as quantitative sensory testing, is needed to more fully investigate the pain mechanisms in these different subgroups. The structural severity of knee and hip OA is not known in this study because the ACR clinical criteria was used and this information would be useful in better understanding these subgroups. Further, generalizability of the study findings are limited to people with symptomatic knee or hip OA and also may be limited by other characteristics of the convenience sample we were able to access for this study. Because a primary goal of the larger research project is to examine fatigue in OA, we excluded people that did not have enough fatigue; however, this was a relatively small portion of the sample (15%). In addition, we included both veterans and non-veterans in this study and this sample comprised about 20% veterans. Veterans are more likely than non-veterans to report chronic joint pain and activity limitations [54] and a larger study would be needed to examine differences between these two groups.

## Conclusions

Among a group of community-dwelling older adults with symptomatic knee or hip OA, we found evidence of three statistically differentiated subgroups that were characterized by differing symptom presentations which may potentially be due to different pain mechanisms. Although further study and replication of the clusters are needed, the heterogeneity of people with symptomatic OA in this sample highlights the need to tailor treatment strategies for symptom management.

## Abbreviations

ANOVA: analysis of variance; BFI: brief fatigue inventory; CES-D: Center for Epidemiological Studies Depression Scale; CNS: central nervous system; MANOVA: multivariate analysis of variance; OA: osteoarthritis; PSQI: Pittsburgh Sleep Quality Index; WOMAC: Western Ontario and McMaster Universities Osteoarthritis Index

## Competing interests

Dr. Clauw's financial disclosures are from Pfizer, Lilly, Forest, Cypress Biosciences, Pierre Fabre, UCB, Jazz Pharmaceuticals, and Merck. Dr. Phillips' financial disclosures are from Merck, United Therapeutics, and Actelion. Drs. Murphy and Williams declare that they do not have competing interests.

## Authors' contributions

The idea for this manuscript arose from a meeting with all co-authors. SLM and AKL conducted the analysis with specific guidance from DAW. SLM provided an initial draft of the manuscript and all coauthors contributed intellectual content, participating in follow-up meetings and in the writing process. SLM acquired the data and assumes responsibility for the integrity of the data as well as the accuracy of the data analysis. All authors read and approved the final manuscript.
